# Decreased BDNF and TrkB mRNA expression in multiple cortical areas of patients with schizophrenia and mood disorders

**DOI:** 10.1038/tp.2014.26

**Published:** 2014-05-06

**Authors:** M T Ray, C Shannon Weickert, M J Webster

**Affiliations:** 1 Stanley Medical Research Institute, Laboratory of Brain Research, Rockville, MD, USA; 2 Trinity Washington University, NE Washington, DC, USA; 3 Schizophrenia Research Institute, Sydney, NSW, Australia; 4 Neuroscience Research Australia, Randwick, NSW, Australia; 5 School of Psychiatry, University of New South Wales, Sydney, NSW, Australia

## Abstract

Abnormalities in brain-derived neurotrophic factor (BDNF)/trkB signaling have been implicated in the etiology of schizophrenia and mood disorders. Patients with schizophrenia, bipolar disorder (BPD) and major depression (MDD) have reduced levels of neurotrophins in their brains when compared with normal unaffected individuals; however, only a few brain areas have been examined to date. Owing to the broad range of symptoms manifested in these disorders, we hypothesized that multiple associative areas of the neocortex may be implicated and that the degree of change in BDNF and trkB−TK+ mRNA expression and the cortical region or layers involved may vary according to Diagnostic and Statistical Manual of Mental Disorders (DSM) diagnosis. We compared BDNF and trkB−TK+ mRNA levels across all layers of the prefrontal cortex (dorsolateral prefrontal cortex, DLPFC), orbital frontal cortex (OFC), anterior cingulate cortex (ACC), inferior temporal gyrus (ITG) and superior temporal gyrus (STG) in four groups: schizophrenia, BPD, MDD and unaffected controls (*n*=60). BDNF mRNA levels were significantly decreased in layers IV and V of DLPFC in schizophrenia patients, in layer VI of ACC in schizophrenia and MDD and in layer VI of ITG in schizophrenia, BPD and MDD. BDNF mRNA levels were also significantly decreased in layer V and/or VI of STG in schizophrenia, BPD and MDD. TrkB−TK+ mRNA levels were only significantly decreased in the cortical layer VI of OFC in BPD. The shared and distinct patterns of neurotrophin transcript reductions, with some specific to each group, may compromise the function and plasticity of distinct cortical areas to various degrees in the different groups and contribute to the range and overlap of symptoms manifested across the diagnoses.

## Introduction

Schizophrenia is a psychiatric disorder characterized by a variety of symptoms including hallucinations, social withdrawal, attention deficits and working memory impairments.^1,2^ Moreover, many of the symptoms that occur in schizophrenia are also present in affective disorders.^3,4^ This broad range of symptoms that manifest in the major mental illnesses is likely to result from shared abnormalities that occur across different neocortical regions, and some diagnostically distinct cortical abnormalities, particularly in associative and limbic areas (such as the dorsolateral prefrontal cortex (DLPFC), superior temporal gyrus (STG), anterior cingulate cortex (ACC) and orbitofrontal cortex (OFC).^5, 6, 7, 8^ The neurotrophic factor brain-derived neurotrophic factor (BDNF), along with its full-length receptor trkB−TK+, regulates synaptic transmission and plasticity in the brain^9^ and are therefore important for normal cognitive functions and are consequently implicated in the pathophysiology of schizophrenia and mood disorders. Abnormalities in BDNF and trkB−TK+ mRNA levels have been reported in various brain regions in those affected by psychiatric disorders and have been replicated in several cohorts. Specifically, BDNF mRNA and protein are decreased in DLPFC in schizophrenia^10, 11, 12, 13, 14^ and in DLPFC, ACC^15,16^ and hippocampus in mood disorders.^17,18^ TrkB−TK+ mRNA abnormalities have been described in the DLPFC,^19,20^ ACC^21^ and hippocampus^17^ in schizophrenia and in DLPFC, ACC^16^ and entorhinal cortex of mood disorders.^17^ Full-length TrkB protein levels are decreased in DLPFC in schizophrenia,^20^ and in the cerebellum in bipolar disorder (BPD).^22^ Several studies have also shown BDNF and TrkB mRNA and protein levels decreased in the DLPFC and hippocampus of depressed suicide victims.^23, 24, 25, 26^ These studies to date, while primarily focusing on the DLPFC and hippocampal regions, nevertheless suggest that the abnormalities in BDNF and trkB−TK+ may be widespread across multiple brain areas; however, it is also possible that neurotrophin abnormalities may vary across cortical regions responsible for different aspects of cognition, emotion and behavior and may differentiate diagnostic categories as defined by Diagnostic and Statistical Manual of Mental Disorders (DSM). On the other hand, it is also apparent that many patients with mental illness do not always fit neatly into a DSM category but display features, both genetic, molecular and symptomatic, that overlap; thus, it is becoming increasing important to understand how the neuropathological changes in the brain of these patients may reflect both the common and different biological and symptomatic features of the disorders.

Here we sought to replicate and extend the previous findings by quantifying BDNF and trkB−TK+ mRNA expression by *in situ* hybridization in the DLPFC, OFC, ACC, STG and inferior temporal gyrus (ITG) of 60 subjects in the Stanley Neuropathology Consortium comprising 15 subjects in each of the four groups: schizophrenia, BPD, major depression (MDD) and unaffected controls. None of the previous studies cited above examined neocortical areas in the Stanley Neuropathology Consortium cohort. By including the three diagnostic groups in the current study we will test for possible diagnostic differences in BDNF or trkB−TK+ expression levels across multiple association cortical areas and cortical layers of the brain. The regional, laminar and diagnostic specificities of BDNF/trkB−TK+ signaling deficits in schizophrenia and mood disorders may compromise the function and plasticity of the different areas to various degrees in the different groups and thereby contribute to the range of symptoms manifested across the diagnostic groups.

## Materials and methods

### Cohort

Dissection of the areas was performed according to structural landmarks using major sulci and gyri from the frozen coronal hemispheres (2- to 3-mm thick) as a guide. Coronal 14-μm-thick slices were sectioned from frozen DLPFC, OFC, ACC, ITG and STG regions of interest (ROIs) cut on a cryostat at the Stanley Medical Research Institute. The cohort consists of 60 subjects divided into four groups: schizophrenia, BPD, MDD and unaffected controls (Table 1). DLPFC sections were taken from Brodmann area 46 (BA46), OFC from BA45 of the orbital gyrus, ACC from BA24 of the cingulate gyrus at the level of the genu of the corpus callosum, ITG sections from BA20 and STG sections from BA22 at the level of the geniculate bodies. Nissl-stained sections were examined to confirm that they contained the relevant areas based on criteria of Brodmann and more recent papers^27, 28, 29^ as described below. Details regarding the post-mortem diagnosis, collection and storage of this cohort have been reported previously^30^ and all the groups were matched for race, sex, age, brain pH, storage interval, post-mortem interval (PMI) and RNA integrity number (RIN; Table 1).

### Riboprobes

The human BDNF complimentary DNA template, 511 bps corresponding to nucleotides 704–1214 (accession no. M61176), was inserted into the Apa1 site of the 2.96-kb Bluescript vector (Stratagene, La Jolla, CA, USA). Human TrkB−TK+ complimentary DNA, 216 bps corresponding to nucleotides 1753–1969 (accession no. U12140) was subcloned into the TA site of the 3.9 -kb pCRII vector (Invitrogen, Carlsbad, CA, USA) and this probe identifies the full-length tyrosine kinase containing version of the TrkB receptor. Probes were purified by DNase treatment, salt and ethanol precipitation followed by ethanol washes. The transcripts recognized with our BDNF and TrkB−TK+ riboprobes were previously confirmed by northern blotting.^10,31^

### *In situ* hybridization

Two sections per subject from each of the four coronal levels containing the five anatomical ROIs were fixed, acetylated, delipidated and dehydrated according to standard protocols.^32^ Slides were then incubated with a radiolabeled antisense probe (labeled with *in vitro* transcription kit (Promega, Madison WI, USA) to a specific activity of ~2.2 × 10^9^) in a hybridization cocktail (300 μl of 5 ng ml^−1^) and allowed to hybridize overnight at 55 °C in humidified chambers. Additional sections were used for the sense stand probes and were radiolabeled to similar specific activities and hybridized and washed along with the adjacent antisense strand sections. Post-hybridization RNAse digestions and stringent washes were performed as previously detailed.^32^ All slides were exposed to Biomax MR (Kodak, Rochester, NY, USA) film for 14 days (BDNF) and 5 days (TrkB−TK+).

### Image analysis

Brain ROIs were analyzed blind to diagnosis using a calibrated densitometric image analysis (NIH Image 1.33) with a 14C standard (nCi g^−1^, Amersham). Three lines (230 μM wide) were placed randomly within the BA of interest but traversing the cortical gray matter perpendicular to the pial surface and optical density measurements were recorded.

Cortical depth percentages of the DLPFC were obtained using the percentage of cortical widths according to the criteria of Rajkowska and Goldman-Rakic^27^. The percentages of DLPFC cortical width were as follows: I (1–10%), II (11–18%), III (19–45%), IV (46–56%), V (57–72%) and VI (73–100%). The cortical depth percentage of the OFC was obtained using the percentage of cortical widths according to the criteria of Rajkowska *et al.*^28^ The percentages of OFC cortical width were as follows: I (1–10%), II (11–20%), III (21–47%), IV (48–61%), V (62–78%) and VI (79–100%). Data for cortical lamina of the ACC were obtained using the percentage of cortical width according to the criteria of Vogt *et al.*^29^ The percentages of ACC cortical width were as follows: I (1–8%), II (9–16%), III (17–39%), Va (40–66%), Vb (67–78%) and VI (79–100%). The percent cortical depth occupied by individual cortical lamina of the ITG and the STG were calculated from Nissl-stained sections. The percentage of full cortical width corresponding to the individual lamina of ITG was as follows: I (1–10%), II (11–20%), III (21–48%), IV (49–58%), V (59–70%), VIa (71–84%) and VIb (85–100%) and for STG: I (1–10%), II (11–25%), III (26–52%), IV (53–62%), V (63–80%) and VI (81–100%). Sampling for individual statistical analysis of each lamina was taken from values within these boundaries.

### Statistical analysis

Statistica 6.0 software (Statsoft, Tulsa, OK, USA) was used to test whether differences existed in BDNF and trkB−TK+ mRNA expression in several anatomically defined cortical regions between the four diagnostic groups. Normal distribution was determined with the Kolmogorov–Smirnov test. Pearson's correlations were performed to examine the relationship between continuous variables (brain pH, storage time, PMI, RIN and age) and BDNF and trkB−TK+ mRNA measures (Supplementary Table S1). If data were not normally distributed (lifetime neuroleptic drug use), a Spearman rank test was performed. If no correlations were detected, an analysis of variance (ANOVA) was used to compare the data; however; when a correlation was observed an analysis of covariance (ANCOVA) was used. If either ANOVA or ANCOVA comparisons reached significance, a Bonferroni *post hoc* test was performed in order to determine the diagnostic group responsible for the significant difference. In addition, outliers in the data set were identified after a close visual boxplot inspection, confirmed via the Grubb's test, that resulted in the removal of one to two individuals from each brain region examined.

We determined whether BDNF or trkB−TK+ mRNA varied according to descriptive categorical characteristics (that is, cerebral hemisphere, gender, suicide, smoking and history of alcohol and/or drug abuse) by using *t*-tests for unequal sample size (Supplementary Table S2). To evaluate the effect that additional medications, antidepressants (ADs) or mood stabilizer (MS) may have on BDNF or trkB−TK+ mRNA expression, all subjects with mental illness were divided into three groups: those not taking the medication at the time of death (AD, *n*=19; MS, *n*=29), those taking the medication at the time of death (AD, *n*=24; MS, *n*=14) and normal controls (*n*=15). ANOVAs were used to examine the effect of these medication categories on BDNF and trkB−TK+ mRNAs in all cortical areas. If an ANOVA reached significance, a *post hoc* least significance test (LSD) was performed to identify the group comparisons responsible for the significant finding.

## Results

### Anatomical mRNA expression pattern of BDNF and trkB−TK+

**Figure 1:**
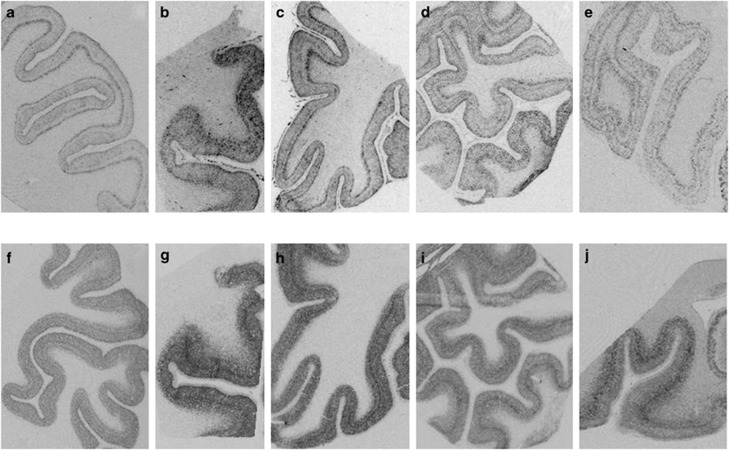
Representative autoradiographic film images of BDNF (**a**–**e**) and trkB−TK+ (**f**–**j**) mRNA hybridization signal in the prefrontal cortex (**a, f**), cingulate cortex (**b, g**), orbital frontal cortex (**c, h**), superior temporal gyrus (**d, i**) and inferior temporal cortex (**e, j**).

BDNF and trkBTK+ mRNA hybridization signal was expressed throughout the gray matter of all cortical areas examined (Figure 1), whereas the sense strand control hybridization did not reveal any discernable signal (Supplementary Figure 1). BDNF mRNA expression was generally more highly expressed in layers II and VI, with limited expression in layer I and subcortical white matter. The laminar pattern of BDNF expression was most pronounced in DLPFC and ITG than in the other cortical areas examined. The trkB−TK+ mRNA signal was also evident in layers II–VI of the gray matter, and while it was more homogenously expressed across layers than BDNF there was a band of increased intensity in the middle cortical layers (deep III and IV) and this band was most prominent in DLPFC and ACC (Figure 1). The mean BDNF and trkB−TK+ mRNA density values for each layer and each area are listed in Supplementary Table S3.

### BDNF and TrkB−TK+ mRNA levels in the cerebral cortex

#### Dorsolateral prefrontal cortex

**Figure 2:**
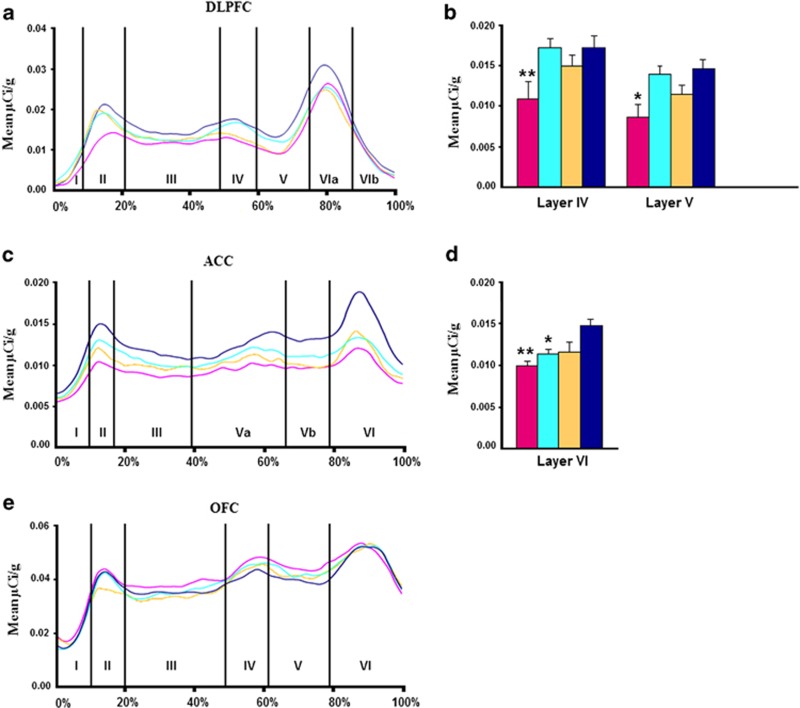
Profiles showing the mean BDNF mRNA levels (μCi g^−1^) for schizophrenia (pink), depression (light blue), bipolar disorder (yellow) and normal controls (dark blue), as a function of distance from the pial surface (**a**) DLPFC, (**c**) ACC, (**e**) OFC. Percentages on the *x* axis refer to proportion of total cortical width occupied by specific lamina. *Post hoc* testing revealed (**b**) schizophrenia group with significantly less BDNF mRNA than controls in layers IV and V of DLPFC and (**d**) schizophrenia and depressed with significantly less BDNF mRNA than controls in layer VI of ACC. *P<0.05, **P<0.01.

Effect of diagnosis: We found a significant main effect of diagnosis for BDNF mRNA levels in cortical layers I, IV and V of the DLPFC (F=4, df=3, 52, *P*=0.01; F=2.93, df=3, 49, *P*=0.04; F=4.03, df=3, 49, *P*=0.01, Supplementary Table S3; Figure 2a). However, Bonferroni *post hoc* testing only showed significant reductions in individuals with schizophrenia compared with controls in layers IV and V (*P*=0.015, 37% reduction; *P*=0.002, 41% reduction; Figures 2a and b). In contrast, we found no diagnostic difference in trkB−TK+ mRNA in the DLPFC by ANCOVAs (all *P*⩾0.57) Supplementary Table S3; Figure 4a).

Correlations with demographic variables: BDNF mRNA levels significantly correlated with PMI (layer II; *r*=−0.30, *P*⩽0.03), pH (layers II–VIb; *r*⩾0.30, *P*⩽0.01), RIN (layers II–VIb; *r*⩾0.26, *P*⩽0.049). TrkB−TK+ mRNA levels correlated with PMI (layer I–VIb; *r*⩾-0.48, *P*⩽0.02), pH (layers I–VIb; *r*⩾0.34, *P*⩽0.01), RIN (layers I–VIb; *r*⩾0.35, *P*⩽0.007, Supplementary Table S1).

#### Anterior cingulate cortex

Effect of diagnosis: There was a significant main effect of diagnosis detected for BDNF mRNA levels in layer VI of the cingulate gyrus (F=5.80, df=3, 52, *P*=0.002; Supplementary Table S3; Figure 2c). Bonferroni testing showed that BDNF mRNA levels were significantly reduced in both schizophrenia (*P*=0.001; 33% reduction) and MDD (*P*=0.03; 24% reduction) compared with controls (Figure 2d). No significant effect of diagnosis was found for trkB−TK+ mRNA level in any cortical layer of the cingulate cortex (all *P*>0.52; Supplementary Table S3; Figure 4b).

Correlations with demographic variables: BDNF mRNA levels did not correlate with any demographic variables in the cingulate, whereas trkB−TK+ correlated with PMI (layers II–Va; *r*⩾-0.36, *P*⩽0.046), pH (layers Va–VI; *r*⩾0.30, *P*⩽0.02) and RIN (layers I–VI; *r*⩾0.31, *P*⩽0.02, Supplementary Table S1).

#### Orbitofrontal cortex

Effect of diagnosis: ANOVAs of BDNF mRNA in the OFC revealed no significant effect of diagnosis (all *P*⩾0.70, Supplementary Table S3; Figure 2e). We did detect a significant effect of diagnosis on trkB−TK+ mRNA levels in cortical layer VI of the OFC (F=2.93; df=3, 51; *P*=0.04; Supplementary Table S3; Figure 4c). Bonferroni *post hoc* showed that trkB−TK+ mRNA levels were significantly reduced in the BPD group (*P*=0.002; 38% reduction) compared with controls (Figure 4d).

Correlations with demographic variables: BDNF mRNA expression levels did not correlate with any demographic variable in the OFC. However, trkB−TK+ mRNA levels correlated with pH (layers I–VI; *r*⩾0.45, *P*⩽0.0004) and RIN (layers I–VI; *r*⩾0.34, *P*⩽0.008, Supplementary Table S1).

#### Superior temporal gyrus

**Figure 3:**
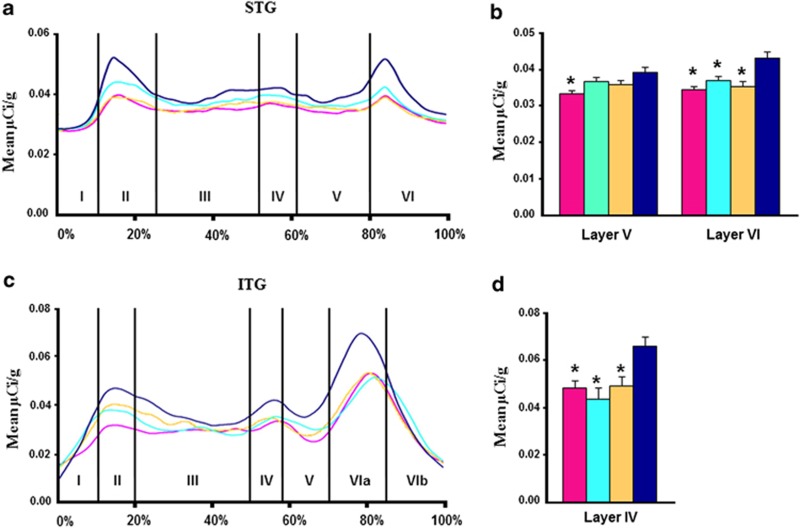
Profiles showing the mean BDNF mRNA levels (μCi g^−1^) for the schizophrenia (pink), depression (light blue), bipolar disorder (yellow) and normal controls (dark blue), as a function of distance from the pial surface (**a**) STG, (**c**) ITG. Percentages on the *x* axis refer to proportion of total cortical width occupied by specific lamina. *Post hoc* testing revealed (**b**) schizophrenia group with significantly less BDNF mRNA than controls in layers V and all three diagnostic groups with significantly less BDNF mRNA than controls in layer VI of STG and (**d**) all three diagnostic groups with significantly less BDNF mRNA than controls in layer IV of ITG. *P<0.01.

**Figure 4:**
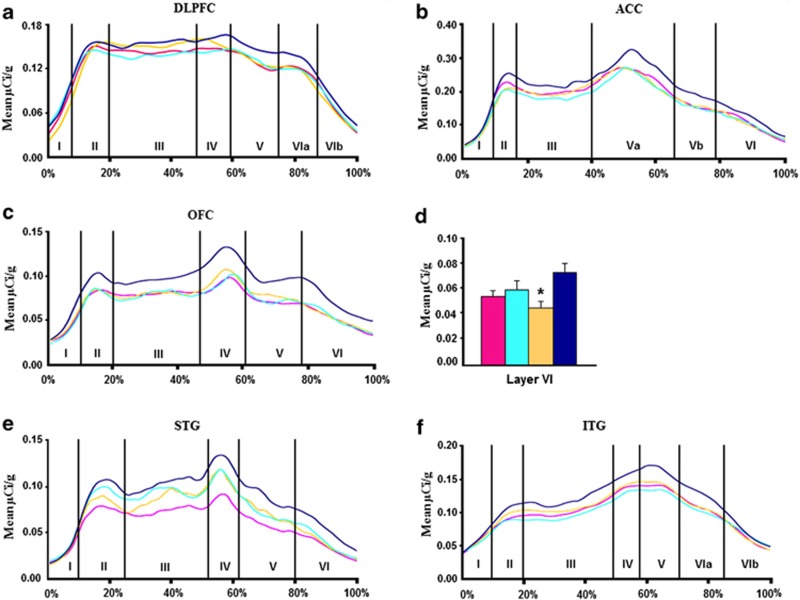
Profiles showing mean trkB−TK+ mRNA levels (μCi g^−1^) for the schizophrenia (pink), depression (light blue), bipolar disorder (yellow) and normal controls (dark blue), as a function of distance from the pial surface (**a**) DLPFC, (**b**) ACC, (**c**) OFC, (**e**) STG, (**f**) ITG. Percentages on the *x* axis refer to the proportion of total cortical width occupied by specific lamina. *Post hoc* testing revealed (**d**) bipolar disorder with significantly less trkB−TK+ mRNA than controls in layers VI. *P<0.005.

Effect of diagnosis: There was a significant main effect of diagnosis detected for BDNF mRNA levels in layers V and VI of the STG (F=2.84, df=3, 53, *P*=0.046; F=5.47, df=3, 52, *P*=0.002, Supplementary Table S3; Figure 3a). Bonferroni *post hoc* tests revealed a significant reduction in BDNF mRNA levels in individuals with schizophrenia (*P*=0.004; 15% reduction) compared with controls in layer V and a reduction in BPD (*P*=0.001; 18% reduction), schizophrenia (*P*=0.0002; 20% reduction) and MDD (*P*=0.01; 14% reduction) compared with controls in layer VI (Figure 3b). No significant effect of diagnosis was detected for trkB−TK+ mRNA in any cortical layer of the STG (all *P*>0.54; Supplementary Table S3; Figure 4e).

Correlations with demographic variables: BDNF mRNA levels were significantly correlated with PMI (layer III; *r*=−0.30, *P*=0.02), pH (layers I, III and VI; *r*⩾0.27, *P*⩽0.04), RIN (layers II–VI; *r*⩾0.33, *P*⩽0.01), whereas trkB−TK+ mRNA was significantly correlated with PMI (layers II–VI; *r*⩾−0.41, *P*⩽0.01), pH (layers II, III and VI; *r*⩾0.28, *P*⩽0.04), RIN (layers II–VI; *r*⩾0.35, *P*⩽0.009, Supplementary Table S1).

#### Inferior temporal gyrus

Effect of diagnosis: We found a significant effect of diagnosis on BDNF mRNA in layer VIa of the ITG by ANCOVA (F=5.79, df=3, 50, *P*=0.002; Supplementary Table S3; Figure 3c). Bonferroni testing showed that BDNF mRNA levels were significantly reduced in BPD (*P*=0.008; 26% reduction), schizophrenia (*P*=0.008; 27% reduction) and MDD (*P*=0.0005; 34% reduction) compared with controls (Figure 3d). There were no significant effects of diagnosis for trkB−TK+ mRNA detected via ANCOVA at any cortical layer in this ROI (all *P*⩾0.40; Supplementary Table S3; Figure 4f).

Correlations with demographic variables: BDNF mRNA levels significantly correlated with pH (layer VIa; *r*=0.28, *P*=0.04), RIN (layer V; *r*=0.27, *P*=0.04). TrkB−TK+ mRNA levels correlated with PMI (layers III–VIa; *r*⩾−0.36, *P*⩽0.02), pH (layers II, IV–VIb; *r*⩾0.30, *P*⩽0.02) and RIN (layers I–VIb; *r*⩾0.31, *P*⩽0.02, Supplementary Table S1).

### Analysis of non-continuous variables on BDNF and trkB−TK+ mRNA levels

BDNF and trkB−TK+ mRNA levels did not typically differ according to gender, hemisphere, suicide or smoking in any of the areas examined in the overall group (Supplementary Table S2). In the OFC, layer V, there was a significant reduction (20%) in BDNF mRNA in male subjects compared with female subjects, although in the STG, layer III, female subjects expressed significantly less BDNF (11%) and trkB−TK+ (20%) than male subjects (*P*⩽0.02, Supplementary Table S2). For BDNF mRNA, there was a significant reduction in the STG in layers II (15%) and III (8%) in the left hemisphere compared with the right (*P*⩽0.03, Supplementary Table S2). There was also a significant reduction in trkB−TK+ mRNA expression in the left hemisphere of the DLPFC (layer I, 19%), OFC (layer I, 17% layer II 20% layer III 20% and layer VI, 26%) and ITG (layer I, 18%) compared with the right (*P*⩽0.04, Supplementary Table S2). Within the subjects with mental illness, individuals with a history of drug and/or alcohol abuse showed lower levels of BDNF mRNA in layer VIb of the ITG (33% *P*<0.04).

### Effect of medications on mRNA levels

**Figure 5:**
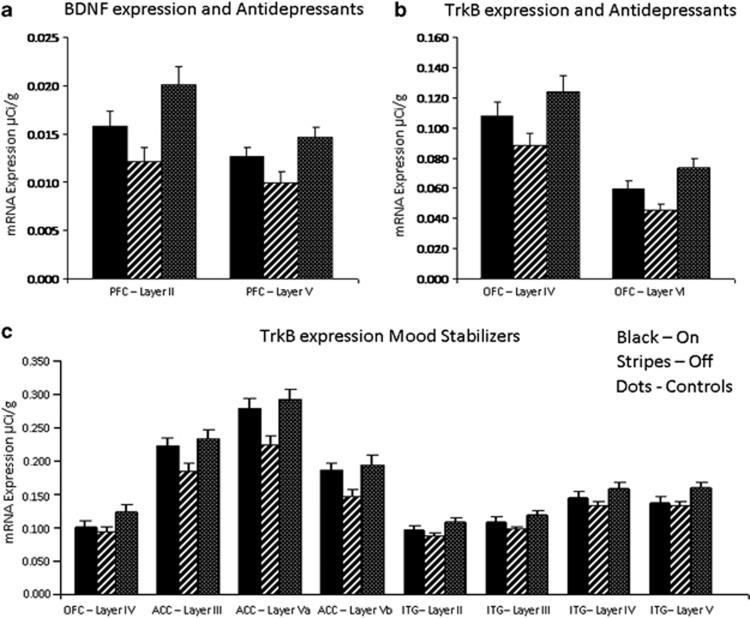
The mean BDNF and trkB−TK+ mRNA levels of individuals on antidepressants (**a, b**) or mood stabilizers (**c**) at the time of death compared with patients free of antidepressants or mood stabilizers at the time of death and to unaffected controls. (**a**) BDNF levels were significantly reduced in DLPFC layers II (*P*=0.006) and V (*P*=0.01) in patients not taking any antidepressants at the time of death as compared with the controls. (**b**) TrkB−TK+ levels were significantly reduced in OFC layers IV (*P*=0.008) and VI (*P*=0.001) in patients not taking any antidepressants at the time of death as compared with controls. (**c**) TrkB−TK+ mRNA levels were significantly reduced in OFC layer IV (*P*=0.01), in ACC layers III (*P*=0.02), Va (*P*=0.002) and Vb (*P*=0.01) and in ITG layers II (*P*=0.0006), III (*P*=0.004), IV (*P*=0.015) and V (*P*=0.01) in the patient group not taking any mood stabilizers at the time of death as compared with the controls groups.

There was no correlation between the continuous variable of lifetime neuroleptic use (in fluphenazine microgram equivalents) and BDNF or trk−BTK+ mRNA levels (Supplementary Table S1). However, to account for the possible effect of AD and MS medications on BDNF and trkB−TK+ mRNA levels, ANOVAs were performed on all areas comparing subjects on ADs at the time of death (*n*=24), patients free of ADs at time of death (*n*=21), and unaffected controls (*n*=15). Whereas ANOVA revealed a significant effect of group in multiple areas, the results that distinguished between the groups on and off ADs were found for BDNF mRNA in DLPFC and trkB−TK+ mRNA in OFC. By LSD analysis, we found BDNF mRNA levels were reduced in layers II and V of the DLPFC in the patient group without recorded use of ADs as compared with the normal control group (F=5.21, *P*=0.002; F=4.76, 0.004, respectively, Figure 5a), whereas the patient group taking ADs was not significantly different from controls (*P*=0.08, *P*=0.21). TrkB−TK+ mRNA levels were significantly reduced in layers IV and VI of OFC in the patient group without recorded use of ADs as compared with normal controls (F=3.83, *P*=0.008; F=6.92, *P*=0.005, respectively, Figure 5b), whereas the group taking ADs was not significantly different from controls (*P*=0.08, *P*=0.07).

A similar ANOVA analysis was conducted to compare those taking MSs at the time of death (*n*=14), those not taking MSs (*n*=29) and normal controls (*n*=15). We found no effect of MS status on BDNF mRNA expression in any region (all F<10.0, all *P*>0.05). In contrast, by LSD analysis, we found that trkB−TK+ levels were significantly reduced in layer IV of OFC (25%) and layers III (17%), Va (24%) and Vb (21%) of ACC in the patient group not taking MSs as compared with controls (F=3.25, *P*=0.01; F=3.92, *P*=0.01; F=6.55, *P*=0.002; F=4.51, *P*=0.01, Figure 5c), whereas the group taking MSs were not significantly different from the controls (*P*⩾0.05). Similarly, trkB−TK+ mRNA levels were significantly lower in layers II (18%), III (17%), IV (19%) and V (19%) of ITG in the patient group not taking MSs as compared with normal controls (F=4.07, *P*=0.0006; F=4.67, *P*=0.004; F=3.22, *P*=0.015; F=3.37, *P*=0.01, respectively; Figure 5c), whereas there was no difference between the group taking MS and the controls (*P*⩾0.05). In each of these cases, the BDNF and trkB−TK+ mRNA levels in the subjects taking the medications are at levels not significantly different from the controls, whereas those not taking the medications are significantly lower than the controls, and thus the mRNA levels may be normalized by the medications.

## Discussion

We show a significant reduction in the expression of BDNF mRNA, particularly in deeper cortical layers and predominantly in schizophrenia in most cortical areas examined (except in the OFC). Specifically, BDNF mRNA levels were significantly decreased in layers IV and V of DLPFC in schizophrenia, in layer VI of ACC in schizophrenia and MDD and in layer VI of ITG in schizophrenia, BPD and MDD. BDNF mRNA levels were also significantly decreased in layer V of STG in schizophrenia and in layer VI of STG in schizophrenia, BPD and MDD. These results corroborate those of earlier studies that indicate low expression of BDNF mRNA in the DLPFC of individuals with schizophrenia to include identification of reductions in a 4th cohort.^10, 11, 12,14^ Further, our studies show that reductions in cortical BDNF are not restricted to the DLPFC nor to just schizophrenia but extend to additional cortical areas and additional groups of patients with major mental illness. This quite widespread reduction in cortical BDNF mRNA in schizophrenia having an impact on many associated cortical areas is in contrast to the lack of significant change in hippocampal BDNF mRNA in schizophrenia found earlier in this same cohort,^17^ suggesting that diagnostic changes in BDNF within the hippocampus are either not present^18,33^ or not as robust as cortical reductions are. Previous studies have shown BDNF mRNA levels to be decreased in deep layers V and VI in DLPFC in schizophrenia;^10,11^ however, these studies also showed significant reductions in BDNF in layer III^10^ and in layers II and III.^11^ Whereas BDNF mRNA levels in our study were also lower in superficial cortical layers in schizophrenia (by 36% in LII and 20% in LIII) as compared with controls, they did not reach statistical significance (Supplementary Table S3). Taken together, these results suggest that, whereas multiple cortical layers can contribute to the overall reduction in DLPFC BDNF mRNA, the reductions in the deeper layers, where BDNF expression is highest, has the largest decrease in schizophrenia. This observation is in line with measurements at the cellular level that suggest that the neurons with a high BDNF expression level (30% of pyramidal neurons in controls) showed the greatest deficit (only 10% of pyramidal neurons in people with schizophrenia).^10^

In contrast to previous studies^11,19,20^ we did not find a decrease in trkB−TK+ mRNA in DLPFC, or in any other area, in schizophrenia. However, our data do show a 12–13% decrease in expression of trkB−TK+ in layers V and VIa in DLPFC in SCH as compared with controls that did not reach statistical significance (Supplementary Table S3). This is somewhat similar to the percent reduction in trkB−TK+ mRNA described in our previous study^20^ that reached a trend level of significance (*P*=0.059). Thus, it is not surprising that we were unable to detect a significant decrease with this smaller cohort (*n*=15 per group) with film-based analysis. Interestingly, the more significant change in trkB receptor levels was previously found in the truncated form of the trkB receptor. The trkB−TK− mRNA levels were significantly increased in the DLPFC in schizophrenia, thereby significantly altering the ratio of trkB−TK+ to trkB−TK− mRNA and protein levels.^20^ The truncated trkB form is capable of binding to BDNF; however, it is not able to mediate the classic neurotrophic response via tyrosine signaling. Thus, an excess of trkB−TK− could contribute to an overall reduction in BDNF/trkB signaling in the DLPFC in schizophrenia.

Whereas trkB−TK+ mRNA levels were not significantly reduced in any other cortical areas in schizophrenia either, we did find that levels tended to be lower in schizophrenia as compared with controls across layers II–VI (16–27%) in OFC, layers III−VI (14–22%) in ACC, layers II–VIa (13–17%) in ITG and layers II–VI (24.5–28%) in STG. These decreases in trkB–TK+ combined with the significant decreases described in various layers for BDNF would indicate that BDNF/trkB signaling is likely to be compromised across multiple cortical areas in schizophrenia.

Previous studies have also shown a decrease in levels of BDNF and/or trkB in cingulate cortex,^21^ hippocampus^17,18,22,33^ and amygdala^34^ in subjects with mood disorder. We find that BDNF mRNA is significantly decreased in layer VI of ACC in MDD, and in layer VI of ITG and STG in both MDD and BPD. We also find that TrkB−TK+ mRNA is significantly decreased in layer VI of OFC in BPD. Thus, just as in schizophrenia, the abnormalities in BDNF/trkB signaling appear to be localized to the deep layers of the cortex and indicate that the neurons in subcortical areas that are supplied to the BDNF by the cortex, including thalamus and striatum, to which they project may have compromised neurotrophic support in these disorders. Many of these subcortical neurons, particularly the GABA neurons of the striatum require BDNF, which is anterogradely transported and released at the axon terminal, for their differentiation and survival.^35, 36, 37, 38^

TrkB−TK+ is expressed homogenously throughout all layers of cortex with a modest increase in middle cortical layers, whereas BDNF synthesis appears to be enriched or exclusive to pyramidal neurons and is more highly expressed in layers V/VI than in more superficial layers I−IV. TrkB−TK+ is expressed almost exclusively in neurons (both large and small) in the cortex and is necessary for activating the second messenger signaling and mediating the neurotrophic effects of BDNF. The decrease in TrkB−TK+ and BDNF mRNA that we find in the deep cortical layers is likely to be due to a downregulation of the mRNA in existing neurons and not due to a reduction in the number of neurons, as previous post-mortem studies in this cohort have revealed no clear change in neuron number or density in any layer of the DLPFC^39^ or OFC^40^ in any diagnostic group. In the ACC, there is an increase in neuronal density in layer V in schizophrenia and BPD and in layer VI in schizophrenia and MDD,^41^ which would also indicate a downregulation of mRNA in existing neurons rather than a loss of neurons. There is evidence for a decrease in neuron soma size in deep layers of the cortex in this cohort^39, 40, 41, 42, 43^ that may reflect the decrease in BDNF particularly since BDNF was initially defined as a molecule necessary to promote cortical neuron somal size and dendritic differentiation during postnatal development and for the maintenance of viable neurons into adulthood.^44,45^ However, in the adult brain the primary functionof BDNF/trkB signaling maybe to potentiate glutamate synaptic transmission and plasticity^46^ and promote synaptic consolidation.^47^ Consequently, the decrease in BDNF that we find may contribute to the abnormalities that are found in markers of synaptic function and plasticity described in the cortex of these cases^48, 49, 50, 51, 52, 53, 54^ and which have also been described in other cohorts.^55, 56, 57, 58^

The current study is reporting on mRNA expression; however, it is likely that the deficits in mRNA levels are reflected in corresponding decreases in protein levels as has been described previously in several studies on human brain.^10,14,20,59^ Likewise, it may be that the blood levels of BDNF in the periphery reflect the brain levels as has been shown in the development and aging of the rat.^60^ Thus, measuring peripheral BDNF levels may provide a biomarker for disease state or provide a marker for treatment response. However, meta-analysis of numerous studies shows that BDNF is generally decreased in the blood of patients with MDD,^61, 62, 63^ in both manic and depressive episodes of BPD^64^ and in schizophrenia.^65^ Thus, with the general decrease in peripheral levels across diagnostic groups in contrast to the specificity of brain deficits to particular areas, lamina and diagnosis, it is unclear as to how informative peripheral levels of BDNF will be.

BDNF and trkB−TK mRNA levels were generally not affected by gender, hemisphere, suicide, history of substance abuse or by smoking. Lifetime intake of neuroleptics was also found to have no correlation with BDNF or trkB−TK+ mRNA levels. Previous human post-mortem studies also found no correlation between antipsychotic use and BDNF and trkB−TK+ mRNA levels.^10, 11, 12,14,17,19^ Rodents treated with typical antipsychotics generally have reduced levels of BDNF expression, whereas those treated with atypicals tend to have increased expression.^66, 67, 68, 69, 70, 71^ The patients in this study were prescribed both types of antipsychotics; therefore, it is not possible to extrapolate effects from the rodent studies. However, we can say that BDNF levels did not correlate with fluphenazine equivalent levels of antipsychotic in our study or in previous postmortem studies.^10, 11, 12,14,17,19^

In contrast to the lack of antipsychotic affect, the use of ADs or MSs did appear to affect the level of BNDF and trkB−TK+ RNA levels in some layers and cortical regions. BDNF mRNA levels were significantly reduced in DLPFC in patients who had not taken AD at around the time of death as compared with patients who had taken them. This suggests that the reductions in BDNF that we found in all three major mental illnesses may have been greater if we had studied only those without a history of AD or MS use. Similarly, TrkB−TK+ mRNA levels were significantly reduced in the cortical regions in patients who had not taken ADs (OFC) or MSs (OFC, ACC and ITC) at around the time of death as compared with patients who had taken them. Thus, those individuals with major mental illness with a history of mood medication often had BDNF and trkB−TK+ mRNA levels comparable to the control group, thus suggesting that this medication may be normalizing an underlying deficiency in BDNF and trkB−TK+ mRNA levels. These studies support the hypothesis that one of the beneficial mechanisms of action for these compounds may include induction of neurotrophin gene expression in widespread areas of the brain.^72^ Several previous studies have also shown a decrease in levels of BDNF and/or trkB in various brain areas in mood disorders,^17,18,21,22,33,34,73^ and just as we show here, many find that patients free of ADs express significantly less BDNF and trkB−TK+ than the patients taking ADs at the time of death.^14,17,33,73^ Moreover, treatment with ADs will upregulate BDNF mRNA and protein in the rodent brain.^74, 75, 76, 77, 78, 79^ Interestingly, the rapid-acting AD effects of ketamine appear to be mediated through an increase in BDNF protein synthesis.^80^ It is likely that these robust AD effects are a consequence of rapid changes in BDNF-mediated synaptic plasticity, which may provide a more direct target for future drug development.

An understanding of the regional, laminar and diagnostic specificities of BDNF and trkB−TK+ deficits in schizophrenia and mood disorders may provide a guide for future therapeutic strategies aimed at this system. Our findings indicate that, whereas BDNF mRNA is decreased in all three diagnostic groups in cingulate and temporal cortices, the frontal cortex is more affected in schizophrenia and the hippocampus is more affected in mood disorders.^17^ This anatomical separation of BDNF/trkB−TK+ signaling deficits may reflect the differences in affective symptoms that manifest between the different mental disorders. The shared patterns of reductions may contribute to the overlap of symptoms manifested across the diagnostic groups. Dissecting the various regional, laminar and cell-type-specific patterns of abnormality that are relevant to the different diagnostic categories may provide a useful guide when considering future therapeutic strategies.

## Figures and Tables

**Table 1:** Stanley Neuropathology Cohort

*Variables*	*Schizophrenia*	*Bipolar disorder*	*Major depression*	*Normal*
Age	44.5±13.1	42.3±11.7	46.5±9.3	48.1±10.7
Sex	9M, 6F	9M, 6F	9M, 6F	9M, 6F
PMI (h)	33.7±15	32.5±16	27.5±11	23.7±10
AP exposure (x10^4^ F-mg-Eq)	5.2	2.1	n.a.	n.a.
Brain pH	6.2±0.25	6.2±0.22	6.2±0.05	6.3±0.24
Race	13C/2A	14C/1AA	15C	14C/1AA
RIN^a^	8.17±0.53	7.92±1.12	7.87±1.20	8.45±0.72
Suicide (yes/no)	4/11	9/6	7/8	0/15

Abbreviations: A, Asian; AA, African American; AP, antipsychotic; C, Caucasian; F, female; F-mg-Eq, fluphenazine milligram equivalent dose; M, male; n.a., not applicable; PMI, post-mortem interval; RIN, RNA integrity number.

Values: means±s.d.

a RIN values were derived from frontal pole screens.
