# Supplementary material for: Decreased BDNF and TrkB mRNA expression in multiple cortical areas of patients with schizophrenia and mood disorders
Source: Transl Psychiatry. 2014 May 6;4(5):e389–. doi: 10.1038/tp.2014.26 (PMC4035720; doi:10.1038/tp.2014.26)
Supplement: Supplementary Figure 1 [file tp201426x1.doc]

Supplementary Figure 1


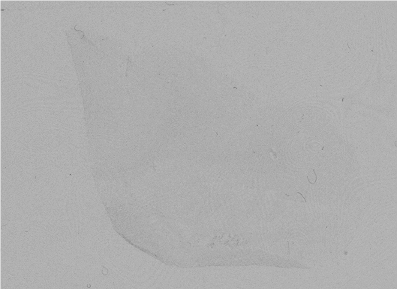

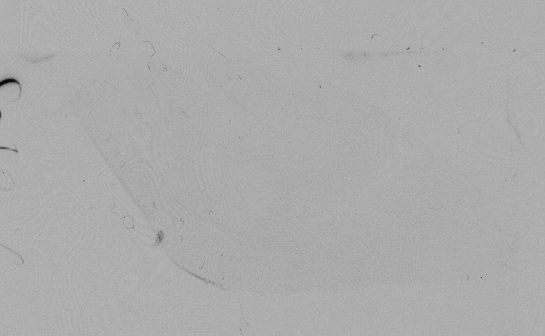


Autoradiographic film images of BDNF (A) and trkB-TK+ (B) sense strand mRNA hybridization signal in representative sections.

A

B
